# Oomycete Soil Diversity Associated with *Betula* and *Alnus* in Forests and Urban Settings in the Nordic–Baltic Region

**DOI:** 10.3390/jof9090926

**Published:** 2023-09-14

**Authors:** Taavi Riit, Michelle Cleary, Kalev Adamson, Mimmi Blomquist, Daiva Burokienė, Diana Marčiulynienė, Jonàs Oliva, Anna Poimala, Miguel Angel Redondo, Gunn Mari Strømeng, Venche Talgø, Leho Tedersoo, Iben Margrete Thomsen, Anne Uimari, Johanna Witzell, Rein Drenkhan

**Affiliations:** 1 Institute of Forestry and Engineering, Estonian University of Life Sciences, F. R. Kreutzwaldi 5, 51006 Tartu, Estonia; taavi.riit@emu.ee (T.R.);; 2 Southern Swedish Forest Research Centre, Swedish University of Agricultural Sciences, Sundsvägen 3, 230 53 Alnarp, Sweden; 3 Nature Research Centre, Akademijos Str. 2, LT-08412 Vilnius, Lithuania; 4 Institute of Forestry, Lithuanian Research Centre for Agriculture and Forestry, Liepų Str. 1, LT-53101 Girionys, Lithuania; 5 Department of Agricultural and Forest Sciences and Engineering, University of Lleida, 25198 Lleida, Spain; 6 Joint Research Unit CTFC–Agrotecnio, 25198 Lleida, Spain; 7 Natural Resources Institute Finland (LUKE), Latokartanonkaari 9, 00790 Helsinki, Finland; 8 Department of Forest Mycology and Plant Pathology, Swedish University of Agricultural Sciences, P.O. Box 7026, 750 07 Uppsala, Sweden; 9 Norwegian Institute of Bioeconomy Research, NIBIO, Høgskoleveien 7, 1433 Ås, Norway; 10 Mycology and Microbiology Center, University of Tartu, J. Liivi 2, 50409 Tartu, Estonia; 11 Department of Geosciences and Natural Resource Management, University of Copenhagen, Rolighedsvej 23, 1958 Frederiksberg C, Denmark; 12 Natural Resources Institute Finland (LUKE), Juntintie 154, 77600 Suonenjoki, Finland; 13 Department of Forestry and Wood Technology, Linnaeus University, 351 95 Växjö, Sweden

**Keywords:** oomycete community, metabarcoding, soil community, soil microbe, *Phytophthora*, plant pathogen

## Abstract

This study aimed to determine the differences and drivers of oomycete diversity and community composition in alder- and birch-dominated park and natural forest soils of the Fennoscandian and Baltic countries of Estonia, Finland, Lithuania, Norway, and Sweden. For this, we sequenced libraries of PCR products generated from the DNA of 111 soil samples collected across a climate gradient using oomycete-specific primers on a PacBio high-throughput sequencing platform. We found that oomycete communities are most affected by temperature seasonality, annual mean temperature, and mean temperature of the warmest quarter. Differences in composition were partly explained by the higher diversity of Saprolegniales in Sweden and Norway, as both total oomycete and Saprolegniales richness decreased significantly at higher longitudes, potentially indicating the preference of this group of oomycetes for a more temperate maritime climate. None of the evaluated climatic variables significantly affected the richness of Pythiales or Peronosporales. Interestingly, the relative abundance and richness of Pythiales was higher at urban sites compared to forest sites, whereas the opposite was true for Saprolegniales. Additionally, this is the first report of *Phytophthora gallica* and *P*. *plurivora* in Estonia. Our results indicate that the composition of oomycetes in soils is strongly influenced by climatic factors, and, therefore, changes in climate conditions associated with global warming may have the potential to significantly alter the distribution range of these microbes, which comprise many important pathogens of plants.

## 1. Introduction

Oomycetes are a group of eukaryotic microbes with lifestyles ranging from saprophytic (living on decaying matter) to pathogenic (attacking live tissue). It is mainly their role as pathogens of trees, crops, and animals that makes understanding the ecology and biology of oomycetes important in order to mitigate losses caused by them [1]. The communities of microbes vary depending on the climatic, edaphic, and host-related variables, which define their habits as saprotrophs, endophytes, pathogens, or parasites [2,3]. Understanding which factors influence oomycete community structure and diversity may help to predict potential shifts in oomycete communities and pathogenic outbreaks, e.g., as a result of certain management activities or in response to global climate change [4]. Many oomycete species, mainly from the genera *Phytophthora* and *Pythium*, are known plant pathogens, which cause significant damage to crops [5,6] and trees [7,8]. While some studies on the biogeography of oomycetes have been published [9,10], knowledge of the factors that shape oomycete communities and their evolution is still very limited.

Oomycete species compositions associated with important broadleaf tree species in natural ecosystems are generally unknown in the Nordic–Baltic region. In this region, alder (*Alnus incana* and *A. glutinosa*) and birch (*Betula pendula* and *B. pubescens*) are among the most common broadleaf trees of economic and ecological importance. Alder trees are a nitrogen-fixing riparian species that provides a habitat for terrestrial and aquatic organisms, nutrient inputs to stream food webs, and stability to riverbanks [11]. Birch represents a significant proportion of the growing stock volume in several countries like Sweden, Finland, Lithuania, and Estonia [12,13,14], and it is an important source material for the production of commercial timber and bioenergy. Additionally, birch is an important species in mixed forests [15] and an alternative to Norway spruce and Scots pine, whose dominance has led to forest health issues in these countries due to numerous pests and pathogens indirectly associated with climate change [16,17,18,19,20]. Diversification of forests is a strategy for mitigating the effects of climate change and assuring that forests continue to provide crucial ecosystem services, including sufficient supply of biomass [21,22,23,24]. In some countries (e.g., Sweden), birch is receiving even wider attention as a potential alternative to spruce and pine [25], and, together with the wider cultivation of birch species, the potential threats to their health should also be considered. Alder species are also widely spread in northern Europe and are commonly used as a source of fire and timber wood. For example, in Estonia, *Alnus incana* makes up approximately 9% and *A. glutinosa* 4% of the total forest area [13].

Multiple studies have analyzed the presence of plant pathogenic oomycete taxa associated with broadleaved tree species in various regions of Europe based on high throughput sequencing (HTS) data, revealing crucial details of possible introduction events and spread of certain pathogens, in particular, species of *Phytophthora* [26,27,28,29,30,31]. However, to our knowledge, no studies have analyzed and compared oomycete communities in alder- and birch-dominated forest and urban sites in the Nordic–Baltic region. Knowledge of these communities would help to establish baseline community profiles that could be compared with future communities that have potentially been altered by climate warming and could present a greater threat to forests due to the combined effects of increased environmental stress and the spread of introduced pathogens.

In this study, we evaluated the effects of site type (forest or urban), tree species (alder or birch), environmental factors, and country on oomycete communities. For this, we tested the following specific hypotheses: (i) oomycete communities differ among the birch- and alder-dominated forests of the five countries; (ii) oomycete communities differ among the forest and urban sites of the five countries; (iii) differences in oomycete communities are significantly associated with climatic factors; (iv) the prevalence of certain *Phytophthora* species is affected by country, site type, and dominant tree species. We hypothesize that any significant differences in oomycete community composition within the relatively small geographic area would indicate a high possibility of significant shifts in these communities in response to future climate change, which could, in turn, lead to increased pathogen damage. Additionally, differences in community composition and species richness among the forest and urban sites would increase the understanding of the impact of human activity and management on oomycetes and the prevalence of potential tree pathogens. To our knowledge, this is the first study aiming to elucidate oomycete community composition and the factors shaping these communities in alder- and birch-dominated soils across a broad area of the Nordic–Baltic region.

## 2. Materials and Methods

### 2.1. Site Selection and Soil Collection

**Figure 1:**
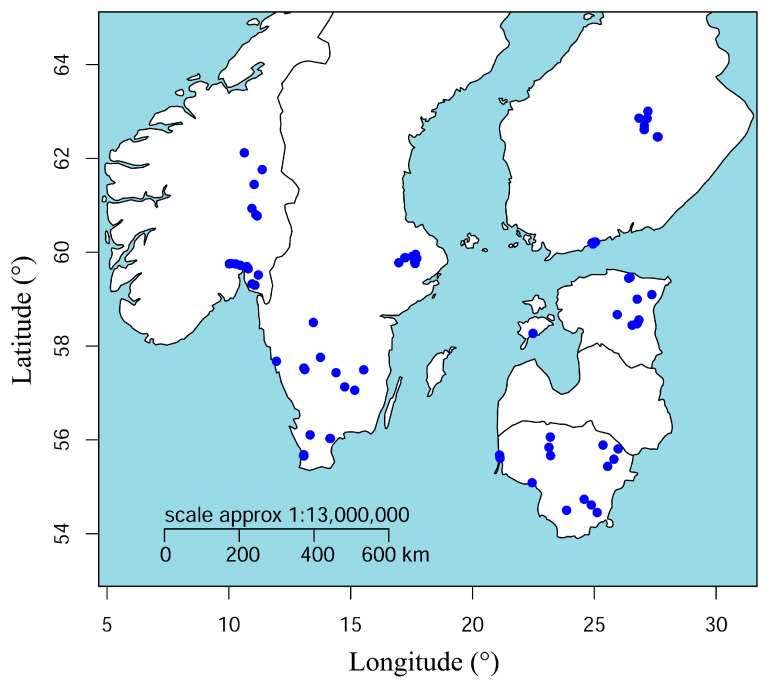
Map presenting the locations of all sampled sites.

Soils around alder and birch trees were sampled from selected forests and urban sites in five countries in the Nordic–Baltic region: Estonia, Finland, Lithuania, Norway, and Sweden (Figure 1; Supplementary Table S1). Sites were selected on the basis of known or suspected presence of *Phytophthora* based on tree-level symptoms (e.g., crown dieback, bleeding lesions on the stem). At each site, ten host trees were selected. The distance between the trees was at least 8 m in the forest sites in order to uniformly sample the whole stand, whereas no restriction regarding the distance between the sampled trees was made for urban sites, where the sampling was targeted to either single or smaller groups of trees. The GPS coordinates, diameter at breast height (DBH), height, and the presence of symptoms, if any, were recorded for each tree.

Following the sampling procedure described by Tedersoo et al. [32], soil was collected from four cardinal locations around each selected tree using a soil core or PVC pipe and trowel. The soil was sampled 0.5–1 m from the root collar (up to 1.5 m for large mature trees) to a depth of between 5 and 20 cm. Tools were cleaned and sterilized after collecting each soil sample to prevent cross-contamination between samples. Soil samples were pooled per sampling site, with each pooled sample comprising approximately 0.5 kg of soil. The samples were then mixed thoroughly in plastic bags and transferred to the lab where they were stored at −20 °C until further processing. In total, 111 soil samples were used for DNA analysis.

### 2.2. DNA Extraction, PCR Amplification and Sequencing

Prior to DNA extraction, 2 g of each soil sample were freeze-dried and crushed using bead beating with two 3.2 mm diameter stainless steel beads (BioSpec Products, Bartlesville, OK, USA) in 2 mL tubes, which were shaken for 5 min at 30 Hz with the Retsch Mixer Mill MM400 (Retsch, Haan, Germany). The DNA was extracted from 2 g of each homogenized soil sample using the MO BIO PowerMax Soil DNA Isolation Kit (MO BIO Laboratories, Inc., Carlsbad, CA, USA).

The oomycete-specific PCR primer ITS1oo [33] and the universal primer ITS4ngs were used to amplify the ITS1/5.8S/ITS2 region of oomycetes present in the soil samples using a reaction mixture comprising 18 µL of PCR grade water, 5 µL of 5x HOT FIREPol Blend Mastermix (10 mM MgCl_2_) (OÜ Solis Biodyne, Tartu, Estonia), 0.5 µL of both primers (20 µM), and 1 µL of the DNA sample. For the negative control, soil DNA was substituted with PCR-grade water, and for the positive control, a DNA sample extracted from a *Phytophthora* pure culture was used. The thermal cycling program used was described in Riit et al. [33]. High-throughput sequencing was carried out on a PacBio Sequel instrument (Pacific Biosciences; Menlo Park, CA, USA) at Molecular Research LP (MR DNA; Shallowater, TX, USA).

### 2.3. Data Analysis

Preliminary processing of the sequencing data was performed at Molecular Research LP (MR DNA; Shallowater, TX, USA) using their proprietary analysis pipeline. Briefly, the barcodes were trimmed, and sequences with ambiguous base calls were removed. Reads that survived denoising were clustered into operational taxonomic units (OTUs) at a 97% similarity level, followed by removal of singleton sequences and chimeras. As part of the Molecular Research LP pipeline, the OTUs were classified using BLASTn against a curated database derived from the GreenGenes, RDPII, and NCBI databases.

The subsequent OTU table was then manually curated to verify the classification. Briefly, OTUs with identities of 75–79%, 80–84%, 85–89%, 90–96%, and >97% were classified at the levels of class, order, family, genus, and species, respectively. In addition, all automatically assigned taxonomic classifications of OTUs were validated by carrying out manual BLASTn searches against the NCBI nucleotide database. When the reference sequence matched more than one taxon with equal similarity, the level of classification was lowered to an unambiguous level.

### 2.4. Statistical Analysis

All statistical analyses were performed using R version 4.1.2 [34]. First, samples with fewer than 200 oomycete reads as well as OTUs represented by a single read were discarded from the dataset. Oomycete OTU richness and Shannon diversity differences between groups of samples were analyzed using analysis of variance (ANOVA) with type 2 sums of squares as implemented in R function *Anova*. We included the total number of reads per sample as a covariate in order to adjust for variance in sequencing depth. Additionally, we included other control factors, such as site type, dominant tree species, or country, when evaluating the effect of a specific factor on oomycete diversity. Pairwise post hoc comparisons were conducted using R function *emmeans* with adjustment for sequencing depth and other control factors as described for ANOVA. Relative abundances were compared similarly to OTU richness and Shannon diversity, but without adjustment for sequencing depth.

Community differences were analyzed based on square root transformed abundances using permutational multivariate analysis of variance (PERMANOVA) as implemented in R function *adonis2,* and subsequent pairwise comparisons were carried out using function *pairwise.adonis2.* PERMANOVA was carried out based on Bray–Curtis dissimilarities with adjustment for sequencing depth by using the total number of reads per sample as an additional independent variable. We also adjusted for additional control factors, such as site type, tree species, and country, when evaluating the effect of a specific factor on oomycete community composition.

Estimated climatic data of 19 variables was downloaded from the WorldClim database (www.worldclim.org; accessed on 18 January 2022) (Supplementary Table S2). We associated OTU abundances rarefied to the lowest per sample read number of 207 reads with climatic variables, longitude, latitude, country of origin, tree species (alder/birch), and site type (forest/park) using distance-based redundancy analysis (dbRDA) based on Bray–Curtis dissimilarities as implemented in R function *dbrda*. Subsequently, to determine the set of variables that best explains changes in oomycete community composition, we used the *dbrda* ordination object as input for automatic stepwise model building using R function *ordistep* with backward variable selection. Significance of the full *ordistep* model and single variables included in the model were determined using R function *adonis2*. Specifically, the explanatory power of the whole model was calculated based on the *adonis2* output by subtracting residual R^2^ from 1.

To determine the set of variables that best explains changes in total oomycete diversity and the diversity of specific oomycete orders, we used lasso regression as implemented in R function *glmnet*. The important variables identified by lasso regression were then used in negative binomial generalized linear model (GLM) building using function *glm.nb* with manual backward variable selection in order to determine optimal models for explaining the differences in OTU richness. Residual plots based on the GLM models were generated using R function *crPlots*.

## 3. Results

### 3.1. The Taxonomic Diversity of Soil Oomycetes

**Figure 2:**
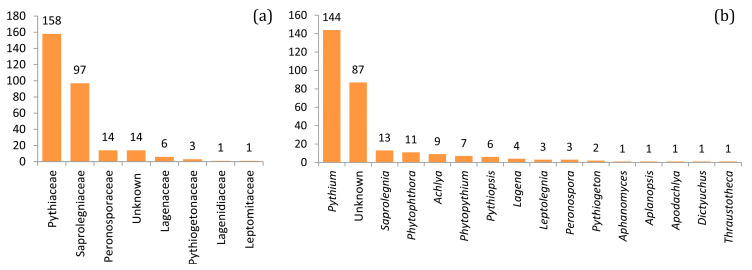
Numbers of oomycete OTUs classified into known (**a**) families and (**b**) genera across all samples.

Sequencing of the 111 soil samples produced 329,562 quality filtered reads that clustered into 2803 OTUs at a 97% similarity rate. After removal of singletons, doubletons, and chimeric sequences, the dataset comprised 195,447 reads divided between 1863 OTUs. After removal of OTUs belonging to other taxa, the final dataset contained 122,840 reads divided between 294 oomycete OTUs, representing 62.9% of all reads and 15.8% of all OTUs, respectively. Of these, 205 OTUs were classified as belonging to known genera and 89 remained unclassified at the genus level (Figure 2). For normalization, 25 samples with less than 200 reads were removed from further analysis. OTUs most prevalent across all sites are presented in Supplementary Figure S1, and the complete OTU table is available at https://dx.doi.org/10.15156/BIO/2483944 (accessed on 8 September 2023).

### 3.2. Phytophthora Species Prevalence in Different Sample Groups

**Figure 3:**
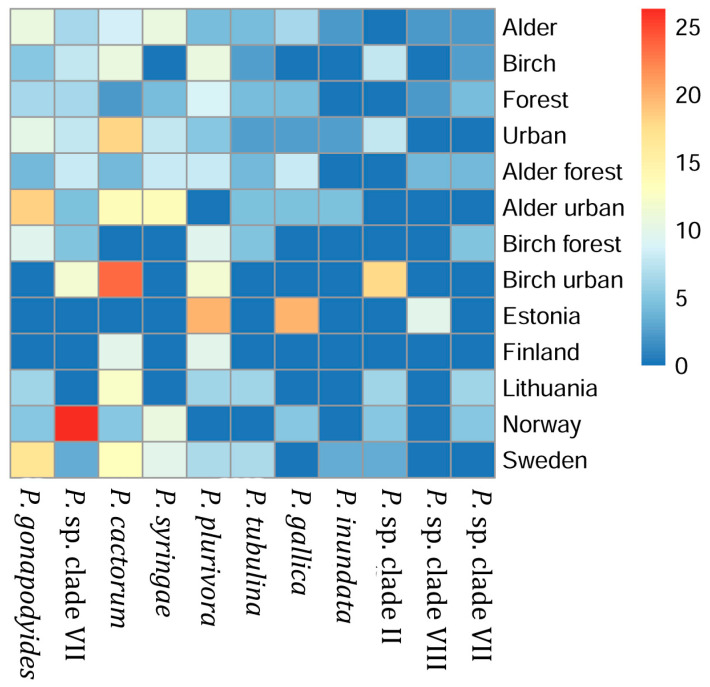
Heatmap showing the proportion (%) of sites in each group where the specific *Phytophthora* species were detected.

A total of 11 OTUs and 935 reads classified as belonging to genus *Phytophthora* were detected across all samples. Seven out of eleven *Phytophthora* OTUs were classified at the species level, matching their respective references with similarities >99.75%, including four OTUs 100% identical to the reference sequences of the species. Out of the most prevalent *Phytophthora* species, *P. cactorum* was detected in eight samples, *P. gonapodyides* in seven samples, and both *P. plurivora* and an unclassified *Phytophthora* species were detected in six samples. Nine samples (11%) contained two or more putative species of *Phytophthora. Phytophthora* species were detected in 37% of alder sites, 41% of birch sites, 35% of forest sites, 44% of park sites, 40% of alder forest sites, 36% of alder park sites, 29% of birch forest sites, and 53% of birch park sites. Prevalence of *Phytophthora* in different groups of samples is shown in Figure 3. Due to limited data, few differences in the prevalence of certain *Phytophthora* species between the different samples were significant. We found that an undetermined species from clade VII [35] (OTU_93) was more prevalent in Norway (26%) compared to Lithuania (0%; *p* = 0.016), Sweden (3%; *p* = 0.024), and Finland (0%; *p* = 0.041). *P*. *plurivora* was more prevalent in birch sites (11%) compared to alder sites (4%; *p* = 0.021), and *P*. *cactorum* was more common in urban sites (18%) than in forest sites (2%; *p* = 0.048).

### 3.3. Oomycete Richness and Composition Differences among Countries

**Figure 4:**
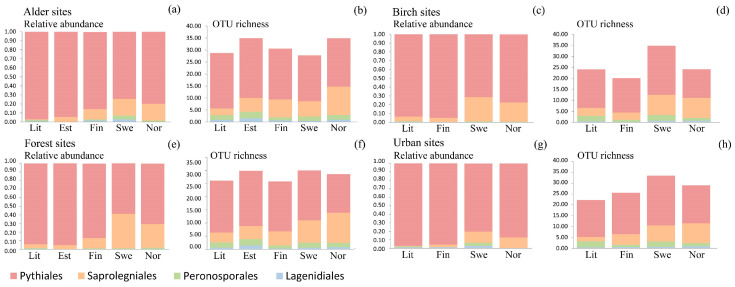
Comparison of oomycete communities among countries based on relative abundances and OTU richness of prevalent orders: (**a**,**b**) alder sites, (**c**,**d**) birch sites, (**e**,**f**) forest sites, and (**g**,**h**) urban sites. Lit—Lithuania, Est—Estonia, Fin—Finland, Swe—Sweden, Nor—Norway.

We found significant differences in community composition among the alder-dominated sites of different countries (R^2^ = 0.14; *p* = 0.003), as well as in the relative abundance of Pythiales (R^2^ = 0.199; *p* = 0.049), with the highest relative abundance in Lithuania (0.97) and the lowest in Sweden (0.74) (Figure 4a, Table 1). OTU richness comparison showed significant differences among the alder-dominated sites of the countries regarding the diversity of Lagenidiales (R^2^ = 0.274; *p* = 0.004), being highest in Estonia (1.5 OTUs/site) and lowest in Sweden (0.45 OTUs/site), and Saprolegniales (R^2^ = 0.264; *p* = 0.002), with the highest richness in Norway (11.72 OTUs/site) and the lowest in Lithuania (2.78 OTUs/site) (Figure 4b, Table 1). Shannon diversity evaluation showed similar trends with significant differences in the diversity of Lagenidiales (R^2^ = 0.212; *p* = 0.040) and Saprolegniales (R^2^ = 0.280; *p* = 0.008), but also in the diversity Pythiales (R^2^ = 0.254; *p* = 0.015), which was highest in Lithuania (1.803) and lowest in Norway (0.931) (Table 1).

There were also significant differences in community composition among the birch dominated sites of the different countries (R^2^ = 0.17; *p* = 0.001), as well as in the relative abundances of Pythiales (R^2^ = 0.210; *p* = 0.021), which was highest in Finland (0.95) and lowest in Sweden (0.71), and Saprolegniales (R^2^ = 0.204; *p* = 0.025), which was highest in Sweden (0.28) and lowest in Finland and Lithuania (0.05) (Figure 4c, Table 1). For the birch dominated sites of the different countries, significant differences were identified in total oomycete diversity (R^2^ = 0.140; *p* = 0.011), which was highest in Sweden (31.2 OTUs/site and lowest in Finland (17.5 OTUs/site), as well as the diversity of Peronosporales (R^2^ = 0.203; *p* = 0.030), which was highest in Sweden (2.63 OTUs/site) and lowest in Finland (0.97 OTUs/site), and Saprolegniales (R^2^ = 0.294; *p* = 0.0002), being highest in Sweden (9.17 OTUs/site) and lowest in Finland (3.27 OTUs/site (Figure 4d, Table 1). In comparison, Shannon diversity analysis revealed significant differences in total oomycete diversity (R^2^ = 0.345; *p* = 0.001) and that of Saprolegniales (R^2^ = 0.384; *p* = 0.0004), with trends similar to those seen in OTU richness, with the exception of Saprolegniales, where highest diversity was found for Norway (0.859) instead of Sweden (0.837) (Table 1).

The forest sites of the different countries differed significantly in oomycete community composition (R^2^ = 0.1576; *p* = 0.003). The differences were significant among the relative abundances of Pythiales (R^2^ = 0.266; *p* = 0.012), being highest in Estonia and lowest in Sweden, and Saprolegniales (R^2^ = 0.256; *p* = 0.015) (Figure 4e, Table 1), which was most prevalent in Sweden (0.40) and least prevalent in Lithuania (0.04). With respect to OTU richness, there were significant differences in the diversity of Lagenidiales (R^2^ = 0.257; *p* = 0.004), being highest in Estonia (1.41 OTUs/site) and lowest in Finland (0.31 OTUs/site), and Saprolegniales (R^2^ = 0.306; *p* = 0.0006) (Figure 4f, Table 1), with the highest diversity found in Norway (11.45 OTUs/site) and lowest in Lithuania (3.66 OTUs/site). Similarly to OTU richness, Shannon diversity varied significantly in Saprolegniales (R^2^ = 0.306; *p* = 0.0006), being highest in Norway (1.250) and lowest in Lithuania (0.171) (Table 1).

The urban sites of the countries also differed significantly regarding oomycete community composition (R^2^ = 0.1469; *p* = 0.002). There were no significant differences in the relative abundances of oomycete orders (Figure 4g), whereas significant differences were found in the OTU richness of Peronosporales (R^2^ = 0.251; *p* = 0.005), with the highest richness found in Lithuania (2.77 OTUs/site) and lowest in Finland (1.13 OTUs/site), and Saprolegniales (R^2^ = 0.081; *p* = 0.014), being most diverse in Norway (9.08 OTUs/site) and least so in Lithuania (2.02 OTUs/site) (Figure 4h). In contrast, comparison of Shannon diversities revealed no significant differences between the countries (Table 1).

### 3.4. Oomycete Richness and Composition Differences among Site Types

**Figure 5:**
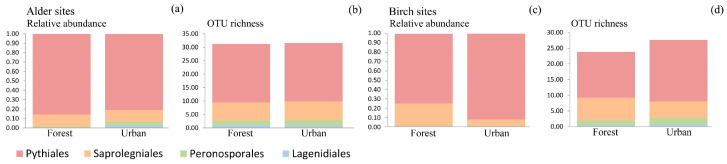
Comparison of oomycete communities among forest and urban sites based on relative abundances and OTU richness of prevalent orders: (**a**,**b**) alder sites and (**c**,**d**) birch sites.

The alder-dominated forest and urban sites did not differ significantly in oomycete community composition, and there were no significant differences in the relative abundances of oomycete orders (Figure 5a, Table 2). Additionally, there were also no significant differences in total OTU richness and in the OTU richness of different orders (Figure 5b, Table 2) or in the Shannon diversities (Table 2).

The birch-dominated forest and urban sites differed significantly in oomycete community composition (R^2^ = 0.080; *p* = 0.002), and there were significant differences in the relative abundances of Pythiales (R^2^ = 0.170; *p* = 0.005) and Saprolegniales (R^2^ = 0.164; *p* = 0.006) (Figure 5c, Table 2). The relative abundance of Pythiales was higher in urban sites (0.92) compared to forest sites (0.75), whereas the relative abundance of Saprolegniales was higher in forest sites (0.24) than in urban sites (0.07). Differences in total OTU richness and in the OTU richness of different orders were not significant (Figure 5d, Table 2), whereas Shannon diversity differed significantly for Pythiales (R^2^ = 0.139; *p* = 0.014), being higher in urban areas (1.176) than in forest (0.681), and Saprolegniales (R^2^ = 0.067; *p* = 0.048), being higher in forests (0.619) compared to urban areas (0.308) (Table 2).

### 3.5. Effect of Climatic and Spatial Variables on Oomycete Community Composition and Species Richness

**Figure 6:**
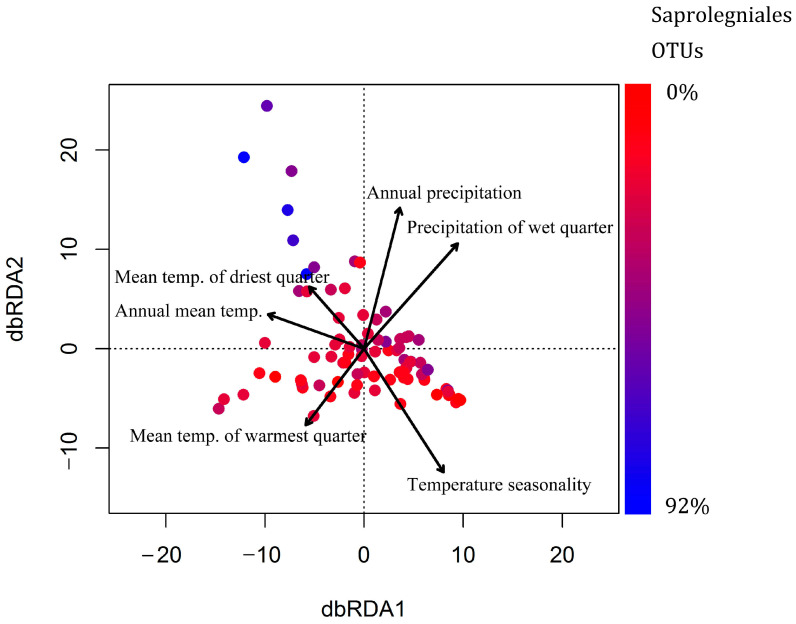
Distance-based redundancy analysis (RDA) ordination plot with climatic variables significantly affecting community composition. Blue and red indicate a higher and lower proportion of Saprolegniales OTUs, respectively. Arrow length indicates the strength of the effect.

The effect of nineteen climatic variables as well as longitude, latitude, country of origin, tree species (alder/birch), and site type (forest/urban) on the composition of oomycete communities was evaluated. The variables were used in automatic stepwise model building using R function *ordistep* to determine the factors most significantly affecting oomycete community composition. The marginal effect of the six significant variables as well as the explanatory power of the full model of climatic variables was assessed using *adonis2* (Table 3), revealing that the best model explained 17.37% of the total variation in the communities. A distance-based redundancy analysis plot was constructed to show the effect of these variables on oomycete composition (Figure 6).

**Figure 7:**
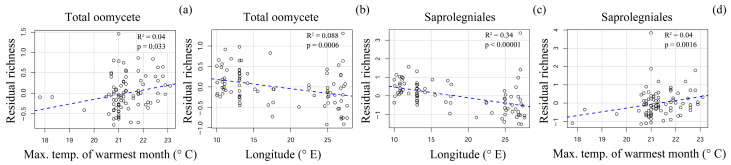
Residual plots of the environmental variables significantly affecting (**a**,**b**) total oomycete OTU richness and (**c**,**d**) Saprolegniales OTU richness.

We also evaluated the effect of climatic variables on total oomycete OTU richness and the richness of the prevalent orders of Saprolegniales and Pythiales. Total oomycete richness was significantly positively affected by maximum temperature of the warmest month (R^2^ = 0.04; *p* = 0.033) and negatively affected by longitude (R^2^ = 0.088; *p* = 0.0006) (Figure 7a,b). Diversity of Saprolegniales was negatively affected by longitude (R^2^ = 0.34; *p* < 0.00001) and positively affected by maximum temperature of the warmest month (R^2^ = 0.04; *p* = 0.0016) (Figure 7c,d). Our analysis did not reveal any factors significantly affecting the diversity of Pythiales in this study range.

## 4. Discussion

The results of community comparisons confirmed our hypotheses that oomycete communities differ among countries, as comparisons among alder-dominated sites, birch-dominated sites, forest sites, and urban sites revealed significant differences. This is in agreement with previous studies on microbial communities in Northern Europe, which have revealed significant differences in composition among sites dominated by the same tree species [36,37,38]. Pairwise comparisons indicated greater differences among geographically more distant countries such as Lithuania and Norway, whereas the differences were less remarkable among neighboring countries, indicating a role of climatic gradients in shaping oomycete communities in the soils similarly to other microbes such as fungi [32,39] and bacteria [40,41,42].

The effect of climatic variables on soil microbial diversity has been well documented in large scale studies [32,43]. It has also been reported that oomycete richness increases with precipitation [3], which can be explained by the water-dependent life-cycle of oomycetes. In this study, variation in total oomycete diversity was relatively low between the groups of samples from different countries, although we did find some evidence of a higher total oomycete diversity in Norway and Sweden. This difference in overall oomycete diversity could be explained by the markedly higher relative abundance and diversity of Saprolegniales in Sweden and Norway, which was significantly inversely correlated with longitude and positively correlated with the maximum temperature of the warmest month, suggesting that the climatic gradients were sufficient to significantly affect the prevalence of certain oomycete groups. Although the effect of latitude on species richness of various taxa is well known due to its direct association with temperature and climate, we hypothesize that across our study area, the longitudinal climatic gradients were more pronounced due to the effects of the warm ocean currents near Norway. The warmth and moisture associated with these currents lead to a longitudinal shift from a more oceanic to more continental climate, which differentially affects various oomycete groups. Notably, longitude explained 34% of the variation in the diversity of Saprolegniales. Although species belonging to Saprolegniales have been reported to have a relatively broad temperature tolerance of 3 to 33 °C [44], they do not grow actively in frozen soil, similarly to other oomycetes [45,46]. Our findings indicate that, compared to Pythiales, species of Saprolegniales are more dependent on the potentially more temperate climate of Norway and southern Sweden, which are more strongly influenced by the warm Norwegian Current. This hypothesis is supported by evidence that occurrences of Saprolegniosis in amphibians appear to be associated with temperate coastal zones [47]. The precise climatic or other conditions that underlie this association between Saprolegniales diversity and longitude across our study range requires further analysis, as no single evaluated climatic variable could reliably explain this trend.

The significant effect of temperature in combination with resulting differences in moisture content on oomycete community composition is in agreement with previously published literature [3,48]. Rojas et al. [48] found that oomycete composition was significantly affected by latitude, longitude, precipitation, and temperature in a soybean production system in North America. Makiola et al. [49] reported that communities of plant pathogens, including those of soil oomycetes, are mainly associated with plant community structure in addition to land use, climate, soil, and geomorphology. Blakney et al. [50] evaluated oomycete communities in *Brassicaceae* field trials and showed that oomycete communities were affected by soil history and chemistry. Our results show that the significant community differences among countries are at least partially explained by the higher diversity and abundance of Saprolegniales in the Scandinavian countries, as the diversity of this group was found to increase markedly at lower longitudes. Our results showed that oomycete communities are affected by both temperature-associated variables, such as temperature seasonality and annual mean temperature, as well as precipitation conditions characterized by precipitation of the wettest quarter and annual precipitation. The effects of temperature and precipitation on oomycete soil composition can be attributed to the water-bound life-cycle of oomycetes and the differential preferences of various groups of oomycetes, such as Pythiales and Saprolegniales. Overall, our findings indicate that changes in climatic conditions are likely to significantly affect oomycete composition and the ranges of certain taxa, as has also been suggested for different fungal species [51,52]. Notably, in this study, the best model comprising the most significant climatic variables only explained 17% of the variation in the oomycete communities. This clearly implies the existence of other highly significant drivers of oomycete communities, which were not considered in this study. In order to more precisely explain and predict the dynamics of oomycete communities in soils, large-scale global studies are required that also consider variables such as those associated with anthropogenic activities, soil composition, and diversity of other organisms in addition to climatic factors [53].

Characterization of *Phytophthora* diversity and potential differences between groups of samples was another aim of this study. Overall, 11 putative *Phytophthora* species were detected across the dataset. We did not find significant differences in the prevalence of *Phytophthora* among any groups of samples from the five countries. In our dataset, *P*. *plurivora* was more prevalent in birch sites than in alder sites, although the species has been reported to infect both *Alnus glutinosa* and *Betula pendula* [54] and has also been reported before in Finnish nurseries [55] and in Norway [56,57]. Notably, this is the first report of *P*. *gallica* and *P*. *plurivora* in Estonia. *Phytophthora cactorum*, which was the most prevalent being detected in eight samples, was found to be more common in urban settings than in forests, which is in agreement with earlier reports [58]. This pathogen has previously been reported in various host trees in Northern Europe, among them both *Alnus* [59] and *Betula* [60], and therefore the relatively high prevalence of *P*. *cactorum* in the analyzed soils of the alder- and birch-dominated sites is not surprising. Although we were not able to confirm the detection of the alder-specific *P. alni* or its subspecies, we did identify an undetermined *Phytophthora* species (OTU_93) belonging to the same clade that was similar to *P*. *formosa* and *P. cambivora* based on sequence identity. As the *P. alni* species complex comprises hybrids that are known to contain ITS sequences representative of more than one species, including those similar to *P. cambivora* [61], it is possible that this OTU represents one or more species of the *P. alni* complex. This hypothesis, however, is not supported by the fact that this species was not found to be more associated with alder than birch, as would be expected. Overall, this species was detected in six samples, with five detections in Norway and one in Sweden, suggesting a potentially higher prevalence in these Scandinavian countries.

The abundance of *Phytophthora* in the analyzed soil samples from alder- and birch-dominated urban or forest sites can be considered relatively low, which is in agreement with a previous report by Bose et al. [62]. *Phytophthora* species were detected in 45 out of 85 samples, which is also similar to findings previously reported by Landa et al. [31]. Given the low detected abundance of *Phytophthora*, it is possible that some of the taxa present in the soil samples remained undetected, potentially leading to underestimation of the prevalence of pathogenic *Phytophthora* in the analyzed soils [63]. Analysis of *Phytophthora* diversity based on high-throughput sequencing of soil samples using PCR primers covering all oomycetes may not be the optimal solution, and using *Phytophthora-*specific primers like those specified in the recent paper by Burgess et al. [64] could possibly promote the recovery of more complete diversity [65].

## 5. Conclusions

Successful adaptation of forestry and urban greening to climate change necessitates a thorough understanding of the biological threats to tree species. As the role of soil oomycetes in tree declines is increasingly recognized, it is critical to understand the patterns of distribution and prevalence of these organisms in the wider environment. With a sampling effort that included alder- and birch-dominated forest and urban sites across Fennoscandian and Baltic countries, our study provides an overview of the regional-scale variation in oomycete communities in Northern Europe. Several ongoing global processes, such as climate warming and intensification of global trade, have been associated with plant disease pressure [66,67] and could result in increasing future damage caused to trees and crops. In order to mitigate potential damage through the timely implementation of management practices, it is necessary to continuously monitor the populations and spread of pathogens in order to improve our understanding of these processes. The results of this study establish a baseline for continued monitoring of the pathogen population in the region in light of potential changes associated with alterations in the climatic conditions accompanied by global trade.

## Figures and Tables

**Table 1:** Differences in mean relative abundance, OTU richness, and Shannon diversity of three prevalent oomycete orders among the countries.

Alder-Dominated Sites		Lit	Est	Fin	Swe	Nor	Significant Pairs
Pythiales	Rel. abund.	1.001	0.981	0.830	0.711	0.822	-
	OTU rich.	23.1	24.9	21.2	19.1	20.2	-
	Shannon div.	1.803	1.195	1.562	0.992	0.931	LN *
Saprolegniales	Rel. abund.	−0.032	0.008	0.142	0.221	0.155	-
	OTU rich.	2.78	5.74	7.42	6.33	11.72	LN **, EN *
	Shannon div.	0.068	0.383	0.384	0.667	1.196	LN **, EN *
Peronosporales	Rel. abund.	0.018	0.006	0.012	0.042	0.018	-
	OTU rich.	2.04	2.80	1.33	1.89	1.99	-
	Shannon div.	0.051	0.298	0.042	0.281	0.123	-
Birch-dominated sites							
Pythiales	Rel. abund.	0.983	-	0.989	0.733	0.726	-
	OTU rich.	17.5	-	15.7	13.0	22.3	-
	Shannon div.	1.094	-	0.795	1.234	0.591	-
Saprolegniales	Rel. abund.	0.005	-	0.011	0.256	0.264	-
	OTU rich.	3.54	-	3.27	9.17	9.10	LS **, FS **, LN **, FN *
	Shannon div.	0.101	-	0.058	0.837	0.858	LS **, FS **, LN **, FN *
Peronosporales	Rel. abund.	0.007	-	0.000	0.008	0.007	-
	OTU rich.	2.553	-	0.965	2.632	1.327	-
	Shannon div.	0.183	-	0.030	0.103	0.019	-
Forest sites							
Pythiales	Rel. abund.	0.947	0.914	0.886	0.602	0.690	LS *
	OTU rich.	19.8	21.1	19.0	19.0	14.7	-
	Shannon div.	1.310	0.904	1.054	1.008	0.609	-
Saprolegniales	Rel. abund.	0.0367	0.089	0.095	0.383	0.292	LS *
	OTU rich.	3.66	4.95	5.36	8.44	11.45	LN ***, EN *
	Shannon div.	0.171	0.344	0.296	0.956	1.250	LN ***, EN **, LS **, FN *
Peronosporales	Rel. abund.	0.011	-0.003	0.015	0.011	0.016	-
	OTU rich.	2.03	2.45	1.15	2.00	1.64	-
	Shannon div.	0.094	0.241	0.034	0.145	0.123	-
Urban sites							
Pythiales	Rel. abund.	0.948	-	0.967	0.827	0.846	-
	OTU rich.	17.0	-	19.1	22.9	17.5	-
	Shannon div.	1.331	-	1.310	1.237	0.823	-
Saprolegniales	Rel. abund.	0.018	-	0.025	0.117	0.138	-
	OTU rich.	2.02	-	4.83	7.42	9.08	LN *
	Shannon div.	0.016	-	0.078	0.590	0.791	-
Peronosporales	Rel. abund.	0.017	-	-0.002	0.034	0.010	-
	OTU rich.	2.77	-	1.13	2.54	1.55	FS *
	Shannon div.	0.286	-	0.028	0.226	0.029	-

*** *p*-adj. < 0.001, ** *p*-adj. < 0.01, * *p*-adj. < 0.05, Lit (L)—Lithuania, Est (E)—Estonia, Fin (F)—Finland, Swe (S)—Sweden, Nor (N)—Norway, Rel. abund.—relative abundance (mean % of total reads), OTU rich.—mean estimated OTU richness, Shannon div.—mean Shannon diversity.

**Table 2:** Differences in mean relative abundance, OTU richness, and Shannon diversity of three prevalent oomycete orders among forest and urban sites.

Alder-Dominated Sites		Forest	Urban	Significance (*p*-Value)
Pythiales	Rel. abund.	0.823	0.915	0.2500 #
	OTU rich.	21.7	21.7	0.9912 #
	Shannon div.	1.29	1.30	0.9579 #
Saprolegniales	Rel. abund.	0.156	0.042	0.1293 #
	OTU rich.	6.73	6.86	0.9349 #
	Shannon div.	0.638	0.441	0.4057 #
Peronosporales	Rel. abund.	0.015	0.023	0.7553 #
	OTU rich.	1.83	2.19	0.2887 #
	Shannon div.	0.114	0.203	0.5013 #
Birch-dominated sites				
Pythiales	Rel. abund.	0.741	0.975	0.0050 **
	OTU rich.	14.6	19.6	0.1336 #
	Shannon div.	0.681	1.176	0.0139 *
Saprolegniales	Rel. abund.	0.249	0.019	0.0062 **
	OTU rich.	7.26	5.28	0.0812 #
	Shannon div.	0.619	0.308	0.0478 *
Peronosporales	Rel. abund.	0.007	0.004	0.4565 #
	OTU rich.	1.58	2.16	0.2236 #
	Shannon div.	0.100	0.067	0.7103 #

** *p*-adj. < 0.01, * *p*-adj. < 0.05, # *p*-adj. > 0.05, Rel. abund.—relative abundance (mean % of total reads), OTU rich.—mean estimated OTU richness, Shannon div.—mean Shannon diversity.

**Table 3:** Marginal effect of variables most significantly affecting oomycete community composition.

Variable	Variance (%)	F Value	*p* Value
Temperature seasonality	5.99	5.66	0.001
Annual mean temperature	5.75	5.43	0.001
Mean temperature of warmest quarter	5.38	5.08	0.001
Precipitation of wettest quarter	4.13	3.90	0.001
Annual precipitation	3.15	2.97	0.009
Mean temperature of driest quarter	2.29	2.16	0.037

## Data Availability

The data presented in this study are openly available in PlutoF repository at https://dx.doi.org/10.15156/BIO/2483944 (accessed on 8 September 2023).

## Supplementary Materials

The following supporting information can be downloaded at: https://www.mdpi.com/article/10.3390/jof9090926/s1, Figure S1: Heatmap of the most prevalent oomycete OTUs that were present in at least 20 sites; Table S1: List of all sequenced soil samples; Table S2: Raw climatic and environmental data of the analyzed soil samples.
