# Supplementary material for: Oomycete Soil Diversity Associated with Betula and Alnus in Forests and Urban Settings in the Nordic–Baltic Region
Source: J Fungi (Basel). 2023 Sep 14;9(9):926. doi: 10.3390/jof9090926 (PMC10532727; doi:10.3390/jof9090926)
Supplement: Supplementary file 1 [file jof-09-00926-s001.zip › Supplementary Figure S1.pdf]

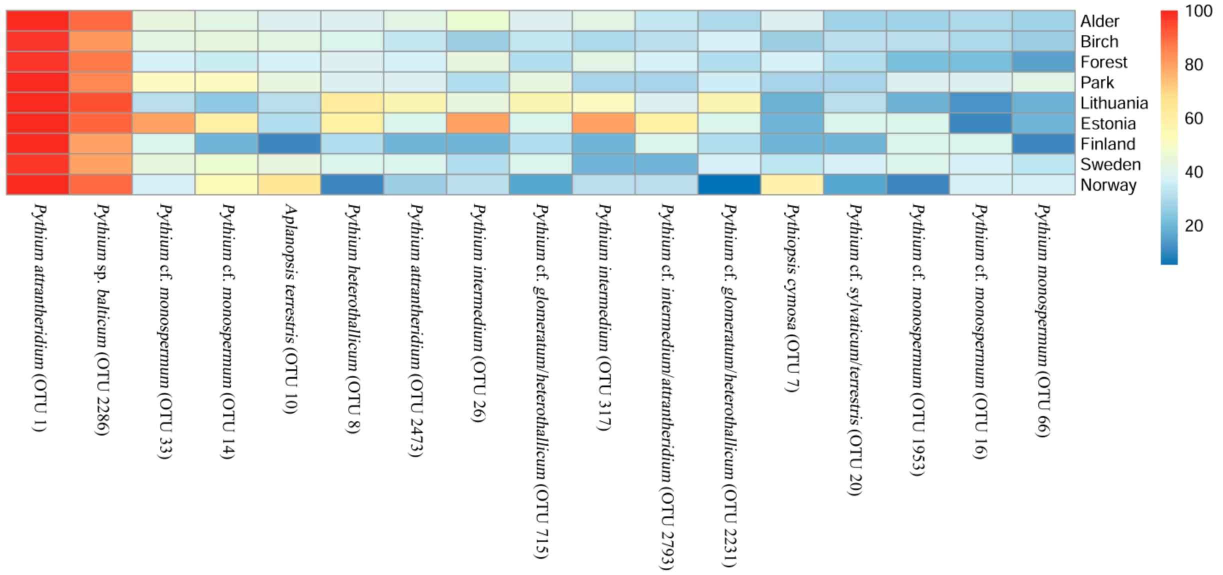

Figure S1. Heatmap of the most prevalent oomycete OTUs that were present in at least 20 sites. Colors indicate the proportion (%) of samples that contained the OTU in each group.
