# Supplementary material for: Oomycete Soil Diversity Associated with Betula and Alnus in Forests and Urban Settings in the Nordic–Baltic Region
Source: J Fungi (Basel). 2023 Sep 14;9(9):926. doi: 10.3390/jof9090926 (PMC10532727; doi:10.3390/jof9090926)
Supplement: Supplementary file 1 [file jof-09-00926-s001.zip › Supplementary Table S1.pdf]

Table S1. List of all sequenced soil samples.

Sample code	Country	Geolocation	Dominant tree	Site type
92	Estonia	58°28'29"N 26°45'16"E	*Alnus* sp.	Forest
170	Estonia	58°26'37"N 26°33'7"E	*Alnus* sp.	Forest
332	Estonia	58°29'43"N 26°46'32"E	*Alnus* sp.	Forest
407	Estonia	59°5'47"N 27°21'40"E	*Alnus* sp..	Forest
467	Estonia	59°27'46"N 26°28'11"E	*Alnus* sp.	Forest
482	Estonia	58°40'15"N 25°56'23"E	*Alnus* sp.	Forest
486	Estonia	59°26'48"N 26°24'27"E	*Alnus* sp..	Forest
495	Estonia	58°16'9"N 22°28'49"E	*Alnus* sp.	Forest
991	Estonia	58°33'27"N 26°49'43"E	*Alnus* sp.	Forest
408	Estonia	58°59'57"N 26°45'29"E	*Alnus* sp.	Park
FIN1	Finland	62°36'48"N 27°3'19"E	*Alnus* sp.	Forest
FIN2	Finland	62°36'49"N 27°2'35"E	*Alnus* sp.	Forest
FIN3	Finland	63°0'14"N 27°11'52"E	*Betula* sp.	Forest
FIN4	Finland	62°50'59"N 27°10'1"E	*Betula* sp.	Forest
FIN5	Finland	62°51'26"N 26°50'2"E	*Betula* sp.	Forest
FIN6	Finland	62°41'41"N 27°3'8"E	*Betula* sp.	Forest
FIN7	Finland	62°27'34"N 27°35'9"E	*Betula* sp.	Forest
FIN8	Finland	62°27'36"N 27°36'52"E	*Betula* sp.	Forest
FIN10	Finland	60°12'30"N 24°58'58"E	*Alnus* sp.	Park
FIN11	Finland	60°12'29"N 24°58'55"E	*Betula* sp.	Park

FIN12	Finland	60°11'51"N 24°54'18"E	*Alnus* sp.	Park
FIN13	Finland	60°10'26"N 24°56'39"E	*Betula* sp.	Park
FIN14	Finland	60°13'07"N 25°02'53"E	*Alnus* sp.	Park
FIN9	Finland	60°12'47"N 24°58'56"E	*Alnus* sp.	Park
LT1	Lithuania	62°36'48"N 27°3'19"E	*Betula* sp.	Forest
LT3	Lithuania	63°0'14"N 27°11'52"E	*Betula* sp.	Forest
LT4	Lithuania	62°50'59"N 27°10'1"E	*Betula* sp.	Forest
LT5	Lithuania	62°51'26"N 26°50'2"E	*Betula* sp.	Forest
LT6	Lithuania	62°41'41"N 27°3'8"E	*Betula* sp.	Forest
LT7	Lithuania	62°27'34"N 27°35'9"E	*Betula* sp.	Forest
LT10	Lithuania	60°12'30"N 24°58'58"E	*Betula* sp.	Forest
LT11	Lithuania	60°12'29"N 24°58'55"E	*Betula* sp.	Forest
LT12	Lithuania	60°11'51"N 24°54'18"E	*Betula* sp.	Forest
LT13	Lithuania	60°10'26"N 24°56'39"E	*Betula* sp.	Forest
LT14	Lithuania	60°13'07"N 25°02'53"E	*Betula* sp.	Forest
LT15	Lithuania	54°29'58"N 23°51'41"E	*Betula* sp.	Forest
LT16	Lithuania	55°05'12"N 22°26'46"E	*Betula* sp.	Forest
LT17	Lithuania	54°36'43"N 24°52'35"E	*Betula* sp.	Forest
LT19	Lithuania	55°40'34"N 21°06'36"E	*Betula* sp.	Forest
LT20	Lithuania	54°36'42"N 24°52'35"E	*Betula* sp.	Forest
TYT	Lithuania	55°39'49"N, 23°12'17"E	Alnus sp.	Forest
ANT	Lithuania	55°48'31"N, 25°58'43"E	*Alnus* sp.	Forest
PAN	Lithuania	55°52'79"N 25°21'23"E	*Alnus* sp.	Forest

JUO	Lithuania	55° 36' 32"N 21°7'54"E	*Alnus* sp.	Forest
ZIO	Lithuania	56°02'99"N 23°11'39"E	*Alnus* sp.	Forest
MOL	Lithuania	55°26'08"N 25°32'41"E	*Alnus* sp.	Forest
UTE	Lithuania	55°34'73"N 25°47'75"E	*Alnus* sp.	Forest
JAG	Lithuania	54°44'02"N 24°34'61"E	*Alnus* sp.	Forest
LT2	Lithuania	62°36'49"N 27°2'35"E	*Betula* sp.	Park
LT8	Lithuania	62°27'36"N 27°36'52"E	*Betula* sp.	Park
LT9	Lithuania	60°12'47"N 24°58'56"E	*Betula* sp.	Park
VOK	Lithuania	54°26'67"N 25°06'59"E	*Alnus* sp.	Park
LT18	Lithuania	55°50'24"N 23°08'17"E	*Betula* sp.	Park
Nor3	Norway	60°46'25"N 11°09'51"E	*Betula* sp.	Forest
Nor5	Norway	59°17'46"N 11°04'28"E	*Betula* sp.	Forest
Nor6	Norway	59°30'53"N 11°12'43"E	*Betula* sp.	Forest
Nor11	Norway	59°45'29"N 10°04'01"E	*Alnus* sp.	Forest
Nor12	Norway	59°45'01"N 10°17'04"E	*Alnus* sp.	Forest
Nor13	Norway	60°47'54"N 11°06'27"E	*Alnus* sp.	Forest
Nor14	Norway	60°55'57"N 10°56'51"E	*Alnus* sp.	Forest
Nor16	Norway	61°26'42"N 11°02'28"E	*Alnus* sp.	Forest
Nor17	Norway	59°19'26"N 10°57'20"E	*Alnus* sp.	Forest
Nor18	Norway	59°43'13"N 10°28'26"E	*Alnus* sp.	Forest
Nor19	Norway	59°41'38"N 10°44'03"E	*Alnus* sp.	Forest
Nor1	Norway	59°45'07"N 10°01'12"E	*Betula* sp.	Park
Nor2	Norway	59°45'34"N 10°04'109"E	*Betula* sp.	Park

Nor4	Norway	61°45'43"N 11°22'05"E	*Betula* sp.	Park
Nor7	Norway	59°45'07"N 10°01'12"E	*Betula* sp.	Park
Nor8	Norway	59°39'56"N 10°45'49"E	*Betula* sp.	Park
Nor9	Norway	59°39'17"N 10°47'19"E	*Betula* sp.	Park
Nor10	Norway	59°38'55"N 10°47'49"E	*Betula* sp.	Park
Nor15	Norway	62°07'06"N 10°38'10"E	*Alnus* sp.	Park
Nor20	Norway	59°40'07"N 10°46'09"E	*Alnus* sp.	Park
B1	Sweden	59°52'4"N 17°42'48"E	*Betula* sp.	Forest
B2	Sweden	59°54'8"N 17°33'28"E	*Betula* sp.	Forest
B3	Sweden	59°53'0"N 17°13'12"E	*Betula* sp.	Forest
B4	Sweden	59°53'0"N 17°14'47"E	*Betula* sp.	Forest
B5	Sweden	59°45'30"N 17°38'46"E	*Betula* sp.	Forest
A3	Sweden	59°54'39"N 17°31'53"E	*Alnus* sp.	Forest
F1	Sweden	58°30'7"N 13°28'7"E	*Betula* sp.	Forest
F2	Sweden	57°25'57"N 14°24'1"E	*Betula* sp.	Forest
F3	Sweden	57°45'33"N 13°45'33"E	*Betula* sp.	Forest
F4	Sweden	57°7'45"N 14°45'19"E	*Betula* sp.	Forest
F6	Sweden	57°3'26"N 15°10'4"E	*Betula* sp.	Forest
F7	Sweden	57°29'33"N 15°32'6"E	*Betula* sp.	Forest
F8	Sweden	57°40'32"N 11°57'11"E	*Alnus* sp.	Forest
F9	Sweden	55°39'28"N 13°4'22"E	*Alnus* sp.	Forest
F10	Sweden	57°40'32"N 11°57'12"E	*Betula* sp.	Forest
F11	Sweden	56°6'23"N 13°20'11"E	*Betula* sp.	Forest

A1	Sweden	59°49'53"N 17°39'39"E	*Alnus* sp.	Park
A4	Sweden	59°46'35"N 16°58'37"E	*Alnus* sp.	Park
A5	Sweden	59°46'59"N 17°37'31"E	*Alnus* sp.	Park
P1	Sweden	57°31'41"N 13°4'40"E	*Alnus* sp.	Park
P2	Sweden	57°31'44"N 13°4'47"E	*Alnus* sp.	Park
P3	Sweden	57°31'45"N 13°4'48"E	*Alnus* sp.	Park
P4	Sweden	57°31'49"N 13°4'59"E	*Alnus* sp.	Park
P5	Sweden	57°31'47"N 13°4'41"E	*Alnus* sp.	Park
P6	Sweden	55°39'26"N 13°4'42"E	*Betula* sp.	Park
P7	Sweden	55°39'27"N 13°4'44"E	*Betula* sp.	Park
P8	Sweden	55°39'26"N 13°4'42"E	*Betula* sp.	Park
P9	Sweden	56°1'46"N 14°10'9"E	*Betula* sp.	Park
P10	Sweden	56°1'43"N 14°9'5"E	*Alnus* sp.	Park
P11	Sweden	57°29'59"N 13°7'8"E	*Alnus* sp.	Park
P12	Sweden	57°30'3"N 13°7'11"E	*Alnus* sp.	Park
P13	Sweden	57°29'56"N 13°7'5"E	*Alnus* sp.	Park
P14	Sweden	57°29'54"N 13°7'4"E	*Alnus* sp.	Park
P15	Sweden	57°29'52"N 13°6'58"E	*Alnus* sp.	Park
P16	Sweden	55°40'57"N 13°4'31"E	*Alnus* sp.	Park
P17	Sweden	55°41'2"N 13°4'36"E	*Alnus* sp.	Park
F12	Sweden	55°39'35"N 13°4'25"E	*Betula* sp.	Park
A2	Sweden	59°57'9"N 17°40'1"E	*Alnus* sp.	Pond
